# Non-Contact Automatic Vital Signs Monitoring of Infants in a Neonatal Intensive Care Unit Based on Neural Networks

**DOI:** 10.3390/jimaging7080122

**Published:** 2021-07-23

**Authors:** Fatema-Tuz-Zohra Khanam, Asanka G. Perera, Ali Al-Naji, Kim Gibson, Javaan Chahl

**Affiliations:** 1 UniSA STEM, Mawson Lakes Campus, University of South Australia, Mawson Lakes, SA 5095, Australia; asanka.perera@mymail.unisa.edu.au (A.G.P.); ali_al_naji@mtu.edu.iq (A.A.-N.); Javaan.Chahl@unisa.edu.au (J.C.); 2 Electrical Engineering Technical College, Middle Technical University, Baghdad 10022, Iraq; 3 Clinical and Health Sciences, City East Campus, University of South Australia, North Terrace, Adelaide, SA 5000, Australia; Kim.Gibson@unisa.edu.au

**Keywords:** heart rate, respiratory rate, NICU, convolutional neural network, signal decomposition

## Abstract

Infants with fragile skin are patients who would benefit from non-contact vital sign monitoring due to the avoidance of potentially harmful adhesive electrodes and cables. Non-contact vital signs monitoring has been studied in clinical settings in recent decades. However, studies on infants in the Neonatal Intensive Care Unit (NICU) are still limited. Therefore, we conducted a single-center study to remotely monitor the heart rate (HR) and respiratory rate (RR) of seven infants in NICU using a digital camera. The region of interest (ROI) was automatically selected using a convolutional neural network and signal decomposition was used to minimize the noise artefacts. The experimental results have been validated with the reference data obtained from an ECG monitor. They showed a strong correlation using the Pearson correlation coefficients (PCC) of 0.9864 and 0.9453 for HR and RR, respectively, and a lower error rate with RMSE 2.23 beats/min and 2.69 breaths/min between measured data and reference data. A Bland–Altman analysis of the data also presented a close correlation between measured data and reference data for both HR and RR. Therefore, this technique may be applicable in clinical environments as an economical, non-contact, and easily deployable monitoring system, and it also represents a potential application in home health monitoring.

## 1. Introduction

A delivery between 37 and 42 weeks of gestation is defined as term pregnancy by the World Health Organization [1]. Preterm birth is defined as any birth prior to 37 weeks of gestation, and it is a priority health issue worldwide. It is projected that more than one in ten of the infants in the world are born prematurely [2]. As preterm infants are not completely developed and have a tendency to have medical conditions that need specialist care, they are often placed into the Neonatal Intensive Care Unit (NICU) immediately after birth [3].

Infants in the NICU have unstable vital signs. According to their particular requirements, specialized medical equipment is used to estimate their physiological condition [4]. The vital signs monitored generally include respiratory rate (RR), heart rate (HR), temperature (T), blood pressure (BP), and oxygen saturation level (SpO_2_) [5,6]. A very high or low heart rate may direct an underlying situation such as pain, infection, or illness. Irregular values of respiratory rate may indicate hypercapnia, hypoxaemia, or acidosis [6,7].

Continuous monitoring of vital signs is usually conducted using different monitoring equipment such as pulse oximeters, electrocardiogram (ECG), respiratory belt transducers, nasal thermocouples, and piezoelectric transducers [8]. Conventional vital sign monitoring equipment involves adhesive transducers or electrodes to be directly attached to the skin. Preterm infant skin is very sensitive and fragile, particularly for those born before 29 weeks of gestational age, when the bond between the dermis and attached sensor may be stronger than that between the epidermis and dermis [9]. As a result, the skin may be damaged. There is also a risk of enabling or introducing an infection [10]. There are numerous established and experimental technologies to remotely monitor an infant’s vital signs, including magnetic induction [11], radar [12], WiFi [13,14], phonocardiograms [15], thermal imaging [16,17], and video camera imaging [18,19].

Magnetic induction-based methods can perceive the impedance changes due to blood and air volume differences caused by the mechanical action of the heart, thorax and diaphragm [20]. This method incorporates a simple arrangement using multiple coils [21] or a single coil integrated into a bed, mattress, or seat [22]. However, the method is highly susceptible to relative movements between coil and body.

Radar- and WiFi-based methods use radio frequency (RF) and exploits wireless signals to monitor vital signs [23]. Standard WiFi devices are used to continuously accumulate the wireless received signal strength (RSS) [24] or channel state information (CSI) [13,14] around a person to detect chest movement. On the other hand, radar-based methods can detect subtle chest movements due to cardiorespiratory activity using the phase shift between the transmitted waves and the reflected received waves from a region of interest (ROI) [25,26].

Thermal imaging-based methods extract vital signs by measuring temperature changes or heat differences due to pulsating blood flow in the main superficial arteries [27,28]. However, both radar and thermal imaging-based approaches are susceptible to noise and motion artefacts and constrain the movement of the subjects [29]. Their relatively low resolution limits the detection range and specificity to one subject. Moreover, these methods need exposed ROI and specialized hardware, making them costly [30]. Additionally, radar-based methods may have biological effects on humans [29].

Video camera imaging extracts vital sings from several regions of the body. The technology can be classified into two main classes: colour-based methods, also known as imaging photoplethysmography (iPPG) [31,32], and motion-based methods [33,34]. The first class exploits skin tone variations owing to cardiorespiratory activity, and the second one relies on cyclic motion of specific regions of the body due to the activity of the cardiorespiratory system. For noncontact monitoring of vital signs, camera imaging-based methods seem to be a promising approach since they are robust, safe, reliable, economical, suitable for long distance and long-term monitoring as well they can measure vital signs from multiple persons simultaneously [29].

In recent years, research in contactless vital signs monitoring using digital video cameras in the near-infrared and visible spectrum (400–1000 nm) has significantly expanded since the technology has become ubiquitous and the cost of digital cameras continues to decrease [29]. It has been revealed that heart rate can be estimated by analyzing the subtle colour variations on the skin surface captured by a video camera [31,32,35,36,37]. Respiratory rate can be estimated by analyzing the movement of certain body parts such as the chest, abdomen or head [34,38]. SpO_2_ can be measured from signals attained from camera imaging using different wavelengths [39,40]. However, all these studies measured the vital signs of the adult population.

Some studies have considered contactless vital signs monitoring of infants using video cameras in a hospital environment. For example, a non-contact monitoring system was first introduced to monitor HR of seven neonates using a low-cost webcam and a non-ambient green light [41]. A manual region of interest (ROI) selection, spatial averaging, independent component analysis (ICA) [42] and power spectral density (PSD) were considered to measure HR. In [43], ambient light was first used to estimate the HR of 19 neonates in a NICU in different challenging conditions. A manually selected ROI was considered as a template to track the global motion of the subject. Fourier analysis was used, and a joint-time-frequency diagram (JFTD) was represented. A camera-based method was developed in [44] for monitoring the HR and RR of seven infants in a NICU using RGB colour magnification and infrared thermography (IRT). A method comparison study was performed on 10 premature babies in the NICU using digital cameras compared with the unit’s cardiorespiratory monitor [45]. To extract HR and RR signals, both colour magnification and motion magnification were used, respectively, based on Eulerian video magnification (EVM). However, EVM carries a high computational cost. Most of the above discussed studies identified confounding factors, such as camera shake, subject movement, limited ROI visibility, noise artefacts, and variable illumination that need to be minimized. An experiment with a similar setting is reported in [46]. They also used RGB and thermal images and showed that the proposed method works when the skin is not visible. More compact wearable motion-based methods have been proposed with increasing success for heart rate [47] and breathing rate [48] monitoring.

To minimize the effect of the changing light conditions and moderate motion artefacts, a robust and efficient method was introduced in [49] using multichannel analysis based on the least-squares method. They reported that the proposed algorithm required 75% less CPU use than ICA. Another clinical study was performed in [50] to monitor the HR of 19 neonates using three CMOS and two LWIR cameras. After selecting the ROI manually, a kernelized correlation filter (KCF) was used to track the ROI. Multiple ROIs were considered to retrieve a signal such as the entire body, face, head, forehead, nose, torso, right arm, left arm, leg and foot. The main challenges addressed by them were artefacts from medical devices, light sources, motion, and the detection and tracking of appropriate regions to retrieve the signal.

In [51], a continuous HR monitoring system was introduced using a webcam where videos of eight neonates were recorded for 30 min each. Another continuous monitoring system was proposed in [52], where two infants were monitored for 40 h by a video camera. A spectral analysis based on auto-regressive modeling and pole cancellation were considered. However, this system was affected by the lighting condition, subject movement, and unclear ROI. However, in all the above discussed studies, the ROI was selected manually.

The respiration of 30 preterm infants was monitored in NICU using a 3-CCD digital camera [53]. In the proposed method, a simple colour-based skin detector was used to segment the ROI. However, colour-based image segmentation is less consistent in a clinical setting. More robust image segmentation is required in order to develop a continuous monitoring system over longer periods of time.

Recent advances in deep learning research have yielded an outline to embed visual features within convolutional neural networks (CNNs) to produce highly precise classifiers in challenging segmentation scenarios [54]. In [55], a multi-task convolutional neural network model was presented to identify the presence of a subject and divide skin regions of the subject to estimate vital signs. This enabled continuous vital signs monitoring that can be performed automatically in challenging clinical situations. The proposed multi-task model had a shared core network with two branches: a patient detection branch employed by means of global average pooling and a skin segmentation branch using a convolutional network. This multi-task CNN model was used in [56,57] to monitor the respiration of five neonatal patients and the vital signs (HR and RR) of 30 preterm infants in the NICU, respectively. The CNN model was extended in [58] by adding a body part detection branch to detect the body parts that are important to estimate the HR, such as the torso, head, and diaper area of the subject. The body part detection branch was executed by means of the Faster R-CNN network. However, computational cost and runtime of CNN are high.

In this work, using visible light video cameras, we remotely monitored both HR and RR of neonates in the NICU using colour and motion-based method, respectively. We trained a baby detection model using the YOLO V3 weights [59] to detect ROI automatically. The YOLO weights were originally trained on the MS COCO dataset [60], which has 80 classes of objects present in it. YOLO works well with multi-scale detections and has shown better accuracy and speed than similar detection models (1000 times faster than R-CNN [61] and 100 times faster than Fast R-CNN [62]). We used a signal decomposition technique to minimize the noise effect using an ensemble empirical mode decomposition (EEMD).

In the literature, the ROI selection of babies for non-contact vital sign monitoring was mainly conducted using manual methods. Vital sign monitoring and acting on irregular vital sign patterns is a real-time requirement. The entire process becomes inefficient when the ROI selection is not automated.

In this study, we (i) proposed an efficient ROI selection method based on a convolutional neural network that could work with different poses of babies in different settings even with unclear regions and (ii) proposed a noise removal method based on a noise-assisted signal decomposition technique to improve the cardiorespiratory signal.

We have found a strong correlation and low error rate between the data measured by the proposed non-contact method and reference data, indicating that video camera imaging can be applied in the NICU and may represent an application to broader contexts such as home health monitoring.

In Section 2, we explain our methods and materials, including study design, the experimental setup for camera imaging-based monitoring in the NICU and the system framework. Subsequently, the results are presented and discussed in Section 3. Finally, this study concludes with the main findings, limitations, and future work in Section 4.

## 2. Methods and Materials

### 2.1. Study Design

A single center cross-sectional observational study was attained at Flinders Medical Centre Neonatal Intensive Care Unit, Adelaide, South Australia. This study was approved by the Southern Adelaide Local Network Research Committee (Protocol no.: HREC/17/SAC/340; SSA/17/SAC/341). After providing a complete explanation of the study measures, written consent from the guardian of the infants was obtained before recording the videos.

We recorded seven infants who were under the monitoring of the regular ECG monitors in the unit. Six infants were preterm (less than 37 weeks gestational age), and one was term. Infants who were not monitored by ECG, those who had unusual characteristics or conditions that may have made them recognizable in publications and those who were likely to be discharged during the data acquisition period were not considered during the experiment.

In this study, for validation purposes, ECG was used as the ground-truth standard for all babies to validate the accuracy of the proposed non-contact technique. The impedance lead of the ECG measures the difference in electrical impedance together with the motion of the chest wall to extract heart rate and respiratory rate. Although it is recognized that ECG has some limitations, such as being influenced by cardiac activity or patient movement [63], it was used for validation purpose to reduce any disruption to the infants or diversion of nursing resources.

### 2.2. Experimental Setup

**Figure 1:**
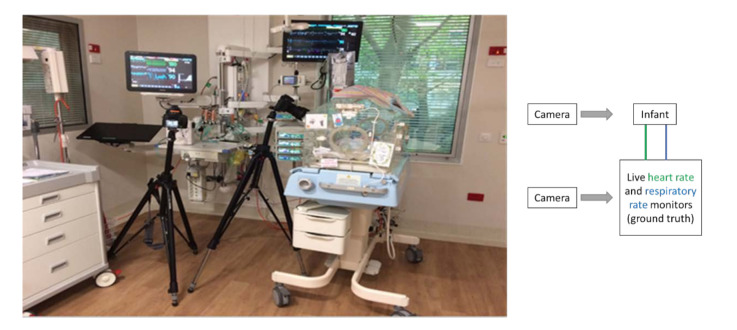
The experimental setup where the data recording was performed. A schematic of the setup is shown to the right of the image. A camera was mounted on a tripod closer to the infant to record the infant body’s heart rate and respiratory rate. Another camera was mounted on a tripod to capture vital signs’ ground truth (shown on the monitor). A schematic diagram on the left of the figure shows an overview of the setting.

The experimental setting is shown in Figure 1, where two digital single-lens reflex (DSLR) cameras were used to record videos, an infant was positioned in the incubator, and the ECG monitor was measuring the vital signs continuously. To record the video of the infant and the ECG monitor, a Nikon D610 with a resolution of 1920 × 1080 and a frame rate of 30 fps, and a Nikon D5300 were used, respectively. The cameras were mounted on tripods. To synchronize data points from the ECG and contactless method, recording from each camera was started simultaneously. The videos were saved in “MOV” format. The digital camera was placed at a distance of 1–2 m away from the patient. We recorded 10 min long videos for each infant. For the experiment, we took 10 s videos when babies were stable, i.e., not moving. For each infant, 5 samples were used.

The most significant challenges we encountered in collecting the videos were the fickle and unstable readings of the hospital monitor attached to the baby. Therefore, we recorded 10 min videos for each child and cut them into 10-s clips when the monitor readings were stable.

### 2.3. System Framework

**Figure 2:**
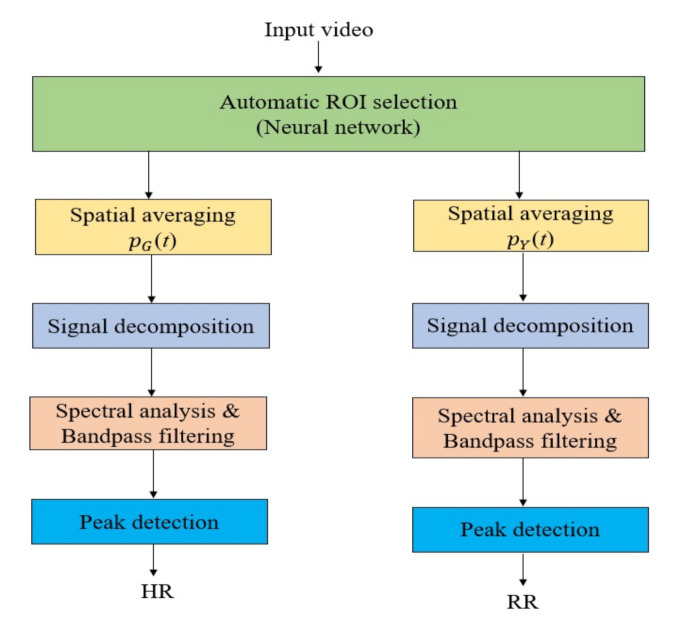
The system framework consists of two branches to detect heart rate and respiratory rate. The input video was processed for automatic ROI detection, and the ROI was processed separately for heart rate and respiratory rate detection.

The overall system includes several image and signal processing techniques, such as automatic ROI selection, spatial averaging, signal decomposition, spectral analysis, band-pass filtering, and peak detection, as shown in Figure 2.

#### 2.3.1. Automatic ROI Selection

In a hospital setting, it is very challenging to record a clear video of babies. Most of the time, their body is occluded with medical instruments or clothes. Datasets for deep learning and testing of algorithms must cover many such variations to be useful in a practical setting.

We collected our images mostly from hospital settings. The images represented different poses of babies in different settings. Images were selected mainly to cover different sleeping positions of babies from different angles while they were fully visible or occluded.

Standard image processing techniques cannot be used for detecting babies in such complex images. There are popular people detector models (YOLO and Mask-RCNN, etc.) available for detecting people of any age, including babies. However, these detectors fail to detect babies in a complex hospital setting as they were trained to detect people in day-to-day situations. Such detectors were trained with very few or no baby images of interest. There are also detectors to detect skeletons and faces [64]. They work well in general situations but fail to detect babies in a hospital setting. This is mainly due to the lack of similar images in the training dataset, occlusions, and lighting conditions.

**Figure 3:**
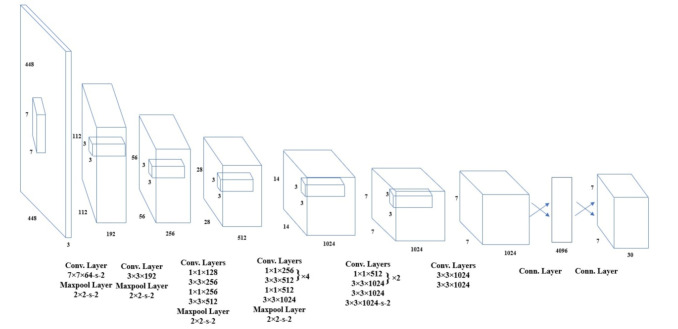
YOLO network architecture (adapted from [65]). The YOLO network has 24 layers followed by two fully connected layers.

**Figure 4:**
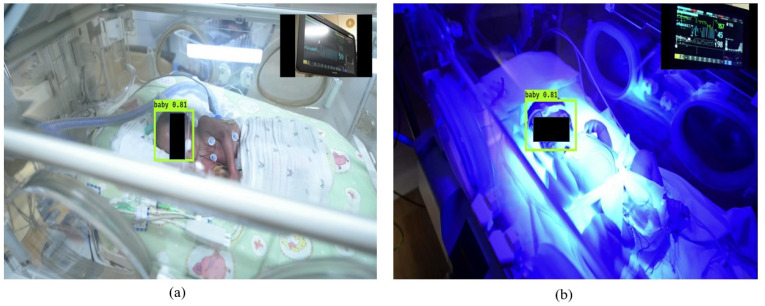
Automatic ROI selection using the YOLO neural network. The detected ROIs were shown in green bounding boxes. (**a**) Infant under normal light, (**b**) infant under blue light.

Therefore, we trained a baby detector using a small dataset gathered from the internet. A total of 473 images were collected for training. The model was trained using the original YOLO V3 weights [59]. The network architecture is illustrated in Figure 3. The YOLO weights were originally trained on the MS COCO dataset [60], which has 80 classes of objects. The YOLO neural network segments the image into regions and predicts bounding boxes and probabilities for each region, as shown in Figure 4. These bounding boxes are weighted by the predicted probabilities. Our detector can detect the face of the baby receiving phototherapy producing a blue light source (Figure 4b).

#### 2.3.2. Spatial Averaging

Colour-based and motion-based methods were used to extract raw cardiac and respiratory signals, respectively. A colour variation in human skin is observed, as haemoglobin in blood absorbs illumination more than surrounding tissue, which may not be visible with the naked eye but can be detected by a video camera [66]. The colour changes reflect the blood volume changes in the microvascular tissue bed under the skin due to variation in pulsatile blood flow during each cardiac cycle. The cardiac signal can be extracted using this principle. Using the green channel of RGB colour space, a raw cardiac signal was extracted by averaging the brightness pixel values of the selected ROI as given in Equation (1). The purpose of this was to minimize noise improve the signal to noise ratio.

(1) $$p_{G}\left(t\right)=\frac{\sum_{i,j\in ROI}P\left(i,j,t\right)}{\left|ROI\right|}$$

where $\left|ROI\right|$ represents the size of the detected ROI and $P\left(i,j,t\right)\;$is the brightness value of a pixel at image location (*i,j*) at a time (*t*). To estimate the cardiac signal, the $p_{G}\left(t\right)\;$signal was chosen as the green channel has the strongest cardiac frequency band compared to the other channels (red and blue) [35]. We found from the literature [67,68] as well as from our experiments that green channel intensity images provide the best estimate for the heart rate. In addition, we skipped the red and blue channels to reduce impacts resulting from skin tone changes and reduce processing time.

Respiratory activity causes cyclic motion in specific body regions such as the head, nostril area, thoracic and abdominal region. In the video recording, spatial variations of intensity values directly indicate this motion. Therefore, the respiratory signal can be measured using this principle. As the video camera captured the video in the RGB colour space, it is required to separate the intensity data from the colour data. Therefore, the RGB colour space was changed to the YIQ colour space. From the Y channel of the YIQ colour space, the raw respiratory signal was measured by averaging the intensity values of the pixels within the selected ROI, as follows:

(2) $$p_{Y}\left(t\right)=\frac{\sum_{i,j\in ROI}P\left(i,j,t\right)}{\left|ROI\right|}$$

where $\left|ROI\right|\;$presents the size of the detected ROI and$\;P\left(i,j,t\right)$ represents the intensity value of a pixel at image location (*i*,*j*) at a time (*t*).

#### 2.3.3. Signal Decomposition

Signal decomposition techniques are applied in biomedical signal processing to separate a temporal signal into a collection of modes of interest. The most appropriate modes are then selected to represent the original signal. The most common signal decomposition techniques are empirical mode decomposition (EMD), ensemble empirical mode decomposition (EEMD), complete ensemble empirical mode decomposition (CEEMD), and complete ensemble empirical mode decomposition with adaptive noise (CEEMDAN).

EMD is a multiresolution signal decomposition technique for a complex and multi-component signal representation developed by Huang et al. [69]. It is commonly used to remove noise artefacts from biomedical signals. The EMD uses the local temporal and structural characteristics of a non-linear and non-stationary signal and adaptively decomposes it into a set of stationary modes in different time scales, called intrinsic mode functions (IMFs). IMF is commonly used to remove noise artefacts from biomedical signals, such as the removal of noise artefacts from ECG data [70], the removal of noise artefacts from electromyogram data [71], the removal of muscle artefacts from electroencephalogram data [72], the removal of tissue artefacts from respiratory signals [73], and the removal of illumination variations from photoplethysmography signal [74].

In order to define a meaningful instantaneous frequency, the corresponding function should be symmetric (symmetry of the upper and lower envelopes) with respect to zero and have the same numbers of zero crossings and extrema. This type of function represents the oscillation mode imbedded in the data and is called an “intrinsic mode function”. Therefore, an IMF can be formally defined as follows.

The decomposition of the original signal, *x*(*t*), into a set of IMFs must occur under two assumptions, as follows: (i) the number of extrema of *x*(*t*) is either equal to the number of zero-crossing or differs at most by one and (ii) the mean values of the envelopes defined by local maxima and local minima are equal to zero.

The signal decomposition based on EMD is defined in the following steps:

1. Identify all peaks (maximum and minimum) of *x*(*t*).

2. Generate the lower and upper envelopes of the peaks through cubic spline interpolation.

3. Calculate the mean value, *m*(*t*), of the lower and upper envelopes point by point.

4. Extract the detail signal, *d*(*t*), by subtracting the mean value *m*(*t*) from *x*(*t*):

*d*(*t*) = *x*(*t*) − *m*(*t*) (3)

5. Verify the properties of *d*(*t*):

a. If *d*(*t*) meets the above two assumptions and becomes a zero-mean process, then it would be the first IMF component of *x*(*t*), named IMF1, and replace *x*(*t*) with the residue:

*r*(*t*) = *x*(*t*) − *d*(*t*) (4)

b. Otherwise, go to step (1) and replace *x*(*t*) with *d*(*t*).

6. Repeat Steps 1 to 5 to obtain the IMF1, IMF2, IMF*N*, where *N* is the number of alterations. The process is stopped when *r*(*t*) becomes a monotonic function, and no further IMF can be extracted.

As a result of the EMD process, *x*(*t*) can be recovered by the following expression:

(5) $$x\left(t\right)=\sum_{i=1}^{N}IMF_{i}+r_{N}\left(t\right)$$

where *N* is the number of IMFs and *r*_*N*_(*t*) is the residue of the signal *x*(*t*).

In this work, we used EEMD, which is a noise-assisted signal decomposition technique proposed by Wu et al. [75], aimed at eliminating the mode-mixing problem caused by the original EMD because of intermittency. It is based on adding white Gaussian noise into the original signal, *x*(*t*), in a controlled manner to obtain the noisy signal, *x*_*m*_(*t*), as follows:

*x*_*m*_ (*t*) = *x*(*t*) + *ω*_*m*_(*t*), *m* = 1, 2, …*L*, (6)

where *ω*_*m*_(*t*) is the *m*th added white noise and *L* is the ensemble number of the EEMD technique. The acquired original signal, *x*_*m*_ (*t*), is then decomposed into a set of IMFs using EMD, and can be recovered as follows:

(7) $$x_{m}\left(t\right)=\sum_{i=1}^{N}IMF_{i,m}+r_{N,m}\left(t\right)$$

(8) $$x\left(t\right)=\frac{1}{L}\sum_{i=1}^{N}\sum_{m=1}^{L}IMF_{i,m}+\frac{1}{L}\sum_{m=1}^{L}r_{N,m}\left(t\right)$$

#### 2.3.4. Spectral Analysis and Band-Pass Filtering

**Figure 5:**
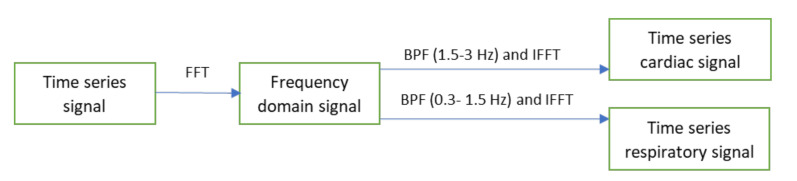
The spectral analysis and band-pass filtering process.

A spectral analysis technique based on the Fast Fourier Transform (FFT) was used to transform the time series cardiac and respiratory signals from the time domain to the frequency domain (see Figure 5). After that, two ideal band-pass filters were applied at 1.5 to 3 Hz, which correspond to the heart pulse range (90–180 beats/min), and 0.3 to 1.5 Hz, corresponding to the breathing range (18–90 breaths/min), to obtain the frequency band of interest. The inverse FFT was then applied to the filtered signals to obtain the time series cardiorespiratory signals.

#### 2.3.5. Peak Detection

The processing of time series cardiorespiratory signals includes calculating peaks and the distance between the consecutive peaks. MATLAB’s built-in ‘findpeaks’ function was used for peak detection. After determining the peaks and their locations (locs), the total cycle length ($C_{L}$) between two peaks can be found using

*C_L_* = mean (diff (locs)) (9)

the number of peaks (M) can be extracted as follows:

(10) $$M=\frac{t}{C_{L}}$$

where *t* represents the video recording length in seconds.

Using the following equation, HR and RR per minute could be calculated:

(11) $$C_{V}=\frac{60M}{t}$$

where *C_V_* represents the calculated value.

## 3. Experimental Results

**Figure 6:**
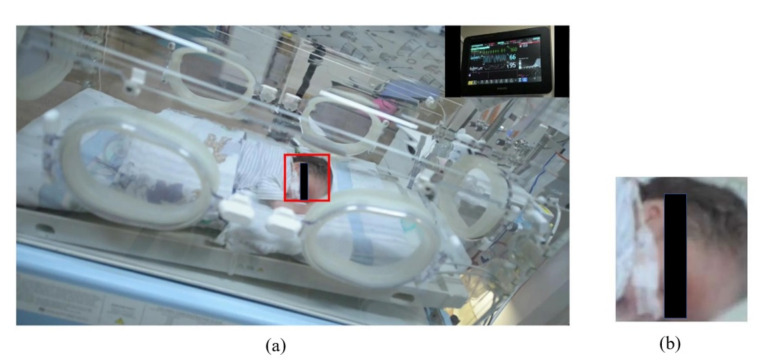
An infant image with detected ROI is shown in (**a**). The corresponding ROI extracted from the original is shown in (**b**).

MATLAB 2020a was used to implement our algorithm and calculate the statistical results. As shown in Figure 6a, the first frame of the video was considered to detect ROI using the YOLO neural network. Figure 6b shows the detected ROI.

**Figure 7:**
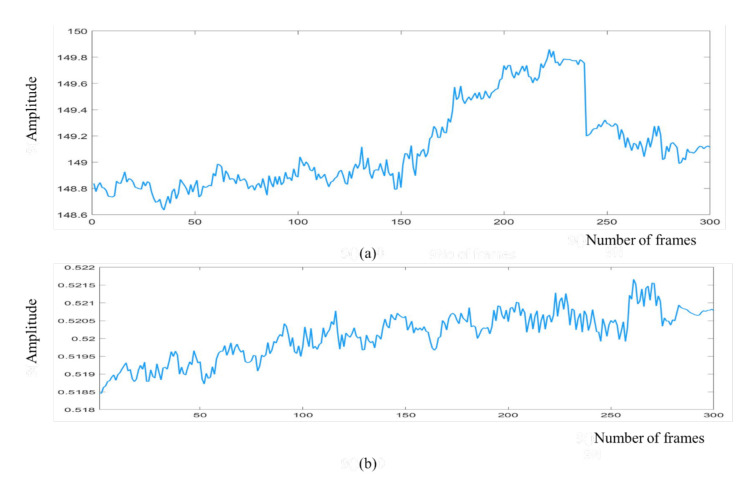
Raw cardiorespiratory signals for 300 frames are shown in the figure. (**a**) Raw cardiac signal, (**b**) raw respiratory signal.

**Figure 8:**
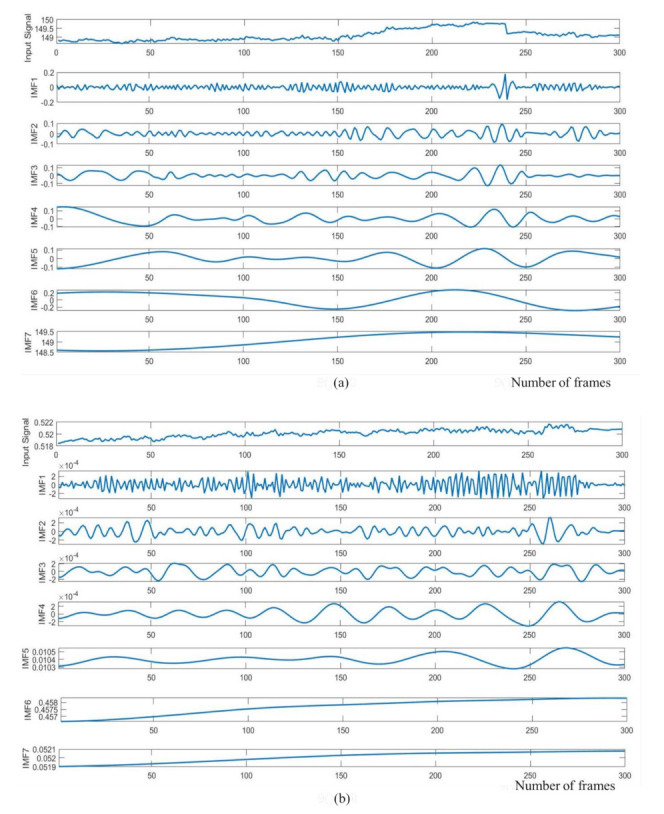
IMF components of the raw cardiorespiratory signals using EEMD technique. (**a**) Cardiac signal (**b**) respiratory signal.

**Figure 9:**
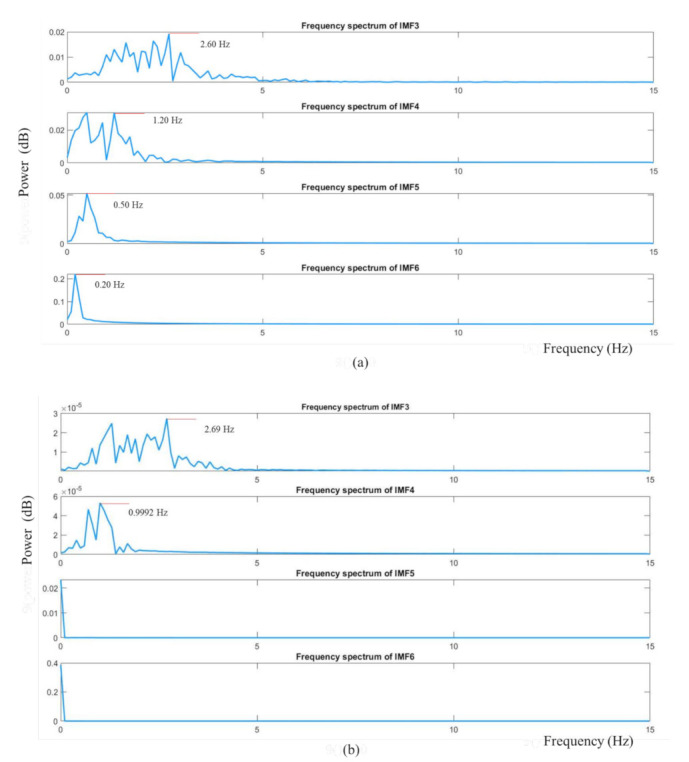
The frequency spectrum of decomposed IMF3, IMF4, IMF5 and IMF6. (**a**) Cardiac signal (**b**) respiratory signal.

We obtained the raw cardiac and respiratory signals after performing spatial averaging over the detected ROI, as shown in Figure 7a,b, respectively. Then, signal decomposition based on EEMD was applied to the raw cardiorespiratory signals. The window length of the EEMD was 10 s. The raw cardiac and respiratory signals were decomposed into IMF1, IMF2, …, IMF7 as presented in Figure 8a,b. To select the best IMF that should be used for calculating the HR and RR, the frequency spectral analysis of the decomposed IMFs was performed using FFT, as shown in Figure 9. Figure 9 shows the spectrum of IMFs 3-6, which have the best frequency bands of interest that fall within the cardiorespiratory range, while other spectra fall outside this range. We selected IMF3 (Figure 8a and Figure 9a) and IMF4 (Figure 8b and Figure 9b) for calculating HR and RR, respectively, as their highest frequencies are close to the frequency of normal HR and RR for infants.

**Figure 10:**
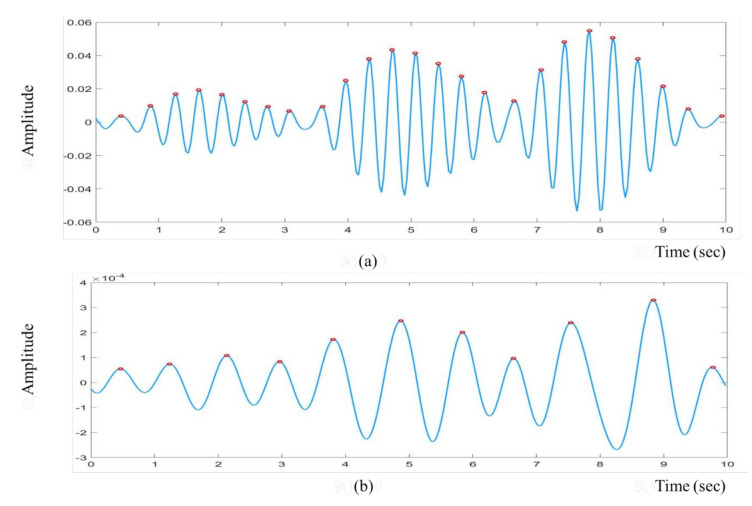
The filtered cardiorespiratory signals are shown in the figure. The red colour markers indicate the peak locations of the filtered signal. (**a**) Filtered cardiac signal, (**b**) filtered respiratory signal.

Figure 10a demonstrates the filtered cardiac signal achieved after FFT, band-pass filtering and inverse FFT. The window length of the FFT was 10 s. From the filtered signal, we calculated the HR by calculating the number of peaks. Similarly, Figure 10b presents the filtered respiratory signal. By calculating the number of peaks, RR could also be calculated from the filtered respiratory signal.

Our ROI selection uses part consists of a neural network. It is the computational heavy component of the proposed solution. Our experiments showed that the ROI selection can process an image of resolution 1920 × 1080 at 1 fps in MATLAB, which is slower than native code implementations would be. In our experiments, the ROI selection and signal processing were conducted separately. ROI selection, together with the signal processing part, can run at roughly 1 fps speed on our test platform (a laptop computer).

**Figure 11:**
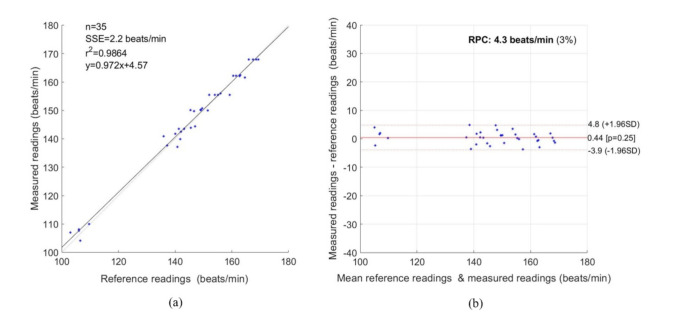
Statistical measurement for HR. (**a**) Correlation Plot, (**b**) Bland–Altman plot.

To evaluate the proposed non-contact system, we considered statistical methods based on the Pearson correlation coefficient (PCC), linear regression, Bland–Altman plot, root mean square error (RMSE) and mean absolute error (MAE). We considered a total sample size, n = 35. Figure 11a shows a strong correlation between the reference and measured data with PCC of 0.9864. As shown in the Bland–Altman Plot in Figure 11b, the reproducibility coefficient (RPC) was 4.3 beats/min (3%), the mean bias was 0.44 beats/min, the lower and upper limits of agreement were −3.9 and +4.8 beats/min. For HR, the RMSE was 2.22 beats/min, and the MAE was 1.80 beats/min.

**Figure 12:**
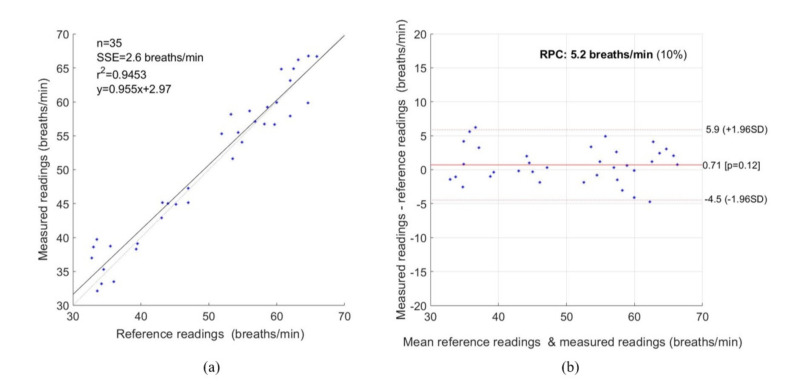
Statistical measurement for RR. (**a**) Correlation Plot, (**b**) Bland–Altman plot.

Figure 12 represents the statistical measurement for RR. As shown in Figure 12a, a strong correlation exists between the reference and measured data with a PCC of 0.9453. Figure 12b represents the Bland–Altman plot with a mean bias of 0.71 breaths/min, a lower and upper limit of agreement of −4.5 and +5.9 breaths/min, and an RPC of 5.2 breaths/min. For RR, the RMSE and MAE were 2.69 and 2.13 breaths/min, respectively.

The standard deviation of heart rate measurement was lower than that of respiratory measurement in this work. This is because the measured heart rate was obtained using the colour-based method. In contrast, the measured respiratory rate was extracted using the motion-based method, which was highly affected by baby movement.

In Table 1, we have compared the Bland

–Altman data for HR with some of the state-of-art methods. Our proposed method showed better results than other methods for all three performance measures compared in the table.

We could conclude that using the proposed system for both HR and RR showed a strong correlation with the reference method with a lower error rate.

## 4. Discussion

In this study, we remotely monitored the vital signs of seven infants in the NICU using video cameras as part of a project to overcome the limitations of contact-based methods. A colour-based method was used to measure HR and a motion-based method was used to calculate RR. Instead of using a manual ROI selection method, an automatic ROI selection method based on a convolutional neural network using the YOLO V3 weights was used to detect ROI. YOLO works well with multi-scale detections and has shown better accuracy and speed than similar detection models (1000 times faster than R-CNN [61] and 100 times faster than Fast R-CNN [62]). Moreover, a signal decomposition technique based on EEMD was also considered to minimize noise artefacts.

In this work, we used EEMD to eliminate the mode-mixing problem caused by the original EMD because of intermittency. However, the EEMD technique may produce noisy IMF components, especially when *L* is relatively low, and may lead to an error in the reconstructed signal. Therefore, more advanced signal decomposition techniques such as CEEMD, CEEMDAN may be used to minimize the limitations of EEMD.

Data collection from infants is a time consuming and challenging process. For this study, we collected a small dataset to validate our proposed method. The data we used for this study comprise a challenging background and varying lighting. Each infant video in our dataset has different setting (clothes, bedsheets, lights, monitoring equipment, etc.). In a hospital setting, it is very challenging to record uncluttered video of infants. Usually, their body is occluded with medical instruments or bedclothes. In addition, the frequent movement of infants is another challenge. Some videos were recorded under poor lighting conditions and one video was recorded while the infant was receiving phototherapy. To improve the reliability of our techniques, we need to experiment with a larger dataset. We are in the process of creating a sufficiently large dataset in this challenging environment.

In this study, ECG was used as the reference for all babies to validate the accuracy of the proposed non-contact technique. Although it is recognized that ECG has some limitations, such as being influenced by cardiac activity or patient movement [63], it was used for validation purposes to reduce any disruption to the infants or diversion of nursing resources.

The most significant challenges we encountered in collecting the videos were the unstable readings of the hospital monitor attached to the baby, which formed our control measurement. Therefore, we recorded 10 min of videos for each child and cut them into 10 s clips when the monitor readings were stable. Longer videos need to be taken and considered in future. The fact that the control data are unstable indicates that the need for sophisticated alternatives to the ECG, such as this, will continue as a major research topic.

Another challenge is that preprocessing, especially filtering, on the IMF signals may cause a change in the waveform and may affect some features of bio signals [76,77,78]. Therefore, more advanced signal processing techniques should be used in the future to improve the robustness of the proposed system and address this limitation.

The experimental results showed a strong correlation with PCC values of 0.9864 and 0.9453 for HR and RR, respectively, and a lower error rate with RMSE values of 2.23 beats/min and 2.69 breaths/min between measured data and ECG data, and MAE values of 1.80 beats/min and 2.13 breaths/min between measured data and ECG data. A Bland–Altman analysis of the data also presented a close correlation between measured data and ECG data for both HR and RR, with a mean bias of 0.44 beats/min and 0.71 breaths/min, and the lower and upper limits of agreement of −3.9 and +4.8 beats/min, and −4.5 and +5.9 breaths/min, respectively. Therefore, not only can this technique be applicable in clinical environments, but it also shows potential for application in home health monitoring due to its non-contact, cost-effective and easily deployable capability.

## 5. Conclusions

In this paper, we measured the HR and RR of seven infants in the NICU based on colour- and a motion-based methods, respectively, using video camera imaging. We used automatic ROI selection based on a convolutional neural network instead of using manual ROI selection. Moreover, to minimize noise artefacts, a signal decomposition technique based on EEMD was also considered. The experimental results showed a strong correlation with PCC values of 0.9864 and 0.9453 for HR and RR, respectively, and a lower error rate with RMSE values of 2.23 beats/min and 2.69 breaths/min and MAE values of 1.80 beats/min and 2.13 breaths/min between measured data and reference data. A Bland–Altman analysis of the data also presented a close correlation between measured data and reference data for both HR and RR with a mean bias 0.44 beats/min and 0.71 breaths/min, and the lower and upper limits of agreement of −3.9 and +4.8 beats/min, and −4.5 and +5.9 breaths/min, respectively. As a result, it can be concluded that this non-contact method has valuable potential as a non-contact, economical and easily deployable monitoring system for use in clinical environments. Still, it also shows a potential application for remote, home health monitoring. However, to calculate vital signs, we had considered the videos when infants were not in motion, and ECG values were also stable. Moreover, the EEMD technique may produce noisy IMF components, especially when *L* is relatively low, leading to an error in the reconstructed signal. In future, to continuously monitor vital signs in the NICU, more advanced signal processing techniques will be required, including all of the practical challenges such as camera movement, subject movement, and illumination variations. Our sample size was also small, so to ensure the system is reliable for real applications, more subjects will need to be considered in future work. We will consider using a dual camera (RGB + thermal) system to further increase our future work reliability.

## Figures and Tables

**Table 1:** Comparison of Bland–Altman data for HR.

Methods	Lower Limit	Upper Limit	Mean Bias
Scalise et al. [41]	−9.79	7.99	0.90
Aarts et al. [43]	−5	+5.5	-
Gibson et al. [45]	−8.3	17.4	4.5
Proposed method	**−3.9**	**+4.8**	**0.44**

## Data Availability

Due to the nature of this research, participants of this study did not agree for their data to be shared publicly, so supporting data is not available.
